# The reduction of ^18^F-FDG uptake ability of tumor tissue after neoadjuvant chemoradiotherapy in locally advanced rectal cancer can effectively reflect the degree of tumor regression

**DOI:** 10.3389/fonc.2022.1037783

**Published:** 2022-12-22

**Authors:** Fengpeng Wu, Xiaoxiao Zhang, Congrong Yang, Kanghua Wang, Linlin Xiao, Chaoxi Zhou, Xinming Zhao, Guiying Wang

**Affiliations:** ^1^ Department of Radiation Oncology, Fourth Hospital of Hebei Medical University, Shijiazhuang, China; ^2^ Department of Radiation Oncology, Hebei Cancer Hospital Chinese Academy of Medical Sciences, Langfang, China; ^3^ Department of Medical Oncology, Affiliated Hospital Of Hebei University, Baoding, China; ^4^ Department of General Surgery, Fourth Hospital of Hebei Medical University, Shijiazhuang, China; ^5^ Department of Nuclear Medicine, Fourth Hospital of Hebei Medical University, Shijiazhuang, China; ^6^ Department of General Surgery, Second Hospital of Hebei Medical University, Shijiazhuang, China

**Keywords:** locally advanced rectal cancer, neoadjuvant chemoradiotherapy (NACRT), 18F-FDG PET-CT, pathological complete regression, near-pathological complete regression, clinical complete regression

## Abstract

**Introduction:**

To evaluate the predictive value of ^18^F-fluorodeoxyglucose positron emission tomography–computed tomography (^18^F-FDG PET-CT) imaging parameters for the response to neoadjuvant chemoradiotherapy (nCRT) in locally advanced rectal cancer (LARC).

**Methods:**

From January 2016 to March 2020, 52 LARC patients who underwent ^18^F-FDG PET-CT scans within 1 week before and 8-9 weeks after nCRT, were enrolled in this study according to a pre-designed screening criteria. After total mesorectal excision (TME) surgery, we assessed tumor response to treatment and analyzed the correlation between imaging parameters obtained from two PET-CT scans and tumor regression status.

**Results:**

Tumor response assessment showed that 13 of 52 patients received good response (GR), including 9 cases with pathological complete regression (pCR) and 4 cases with near-pathological complete regression (near-pCR). We also found that the maximum standard uptake value after nCRT (post-SUVmax), the response index (RI), the mean standard uptake values after nCRT (post-SUVmean), and the ratio of tumor SUVmean to liver SUVmean after nCRT (post-Ratio), were correlated with GR and pCR. Among these parameters, post-SUVmax and RI had a near-strong correlation with pCR (r_s_= -0.58 and 0.59, respectively), and also had a strong correlation with GR (r_s_ = -0.7 and 0.63, respectively). Further ROC analysis showed that post-SUVmax and RI had higher values in predicting whether patients could achieve GR and pCR after nCRT, and the area under the curve (AUC) of both were greater than 0.9. The positive predictive values (PPVs) and negative predictive values (NPVs) of post-SUVmax for GR were 80.01% and 97.3%, and for pCR were 66.68% and 97.5%, respectively. The PPVs and NPVs of the RI values for GR were 84.61% and 94.87%, and for pCR were 69.24% and 100%, respectively.

**Conclusion:**

For LARC patients, the analysis of imaging parameters such as post-SUVmax and RI, which can reflect the changes of ^18^F-FDG uptake capacity of tumor tissues before and after nCRT, is of great value for predicting the response of patients to neoadjuvant therapy and guiding the selection of subsequent treatment strategies.

## Introduction

Neoadjuvant chemoradiotherapy (nCRT) combined with total mesorectal excision (TME) has been widely used as a standard treatment for locally advanced rectal cancer (LARC) patients. However, the follow-up treatment strategy for patients with good response (GR) after nCRT is still controversial. From the perspective of local radical treatment, TME is undoubtedly the most thorough treatment. However, there are some unavoidable problems with this treatment, especially for patients whose primary lesion is adjacent to the anorectal ring plane, even though clinical complete regression (cCR) is obtained, it is difficult to avoid the implementation of Miles operation. In addition, postoperative complications such as intestinal leakage, sepsis, abscess, wound healing difficulties, and cardiopulmonary complications usually increase significantly in patients who have received radical radiotherapy.

At present, the concept of personalized therapy is receiving more and more attention, and the method of screening the group suitable for sphincter retention therapy from the patients with GR after nCRT is increasingly emerging. Watch and Wait (W&W) strategy and local excision (LE) strategy are the focus of subsequent treatment of patients with cCR and near clinical complete response (near-cCR). However, clinical data show that the local recurrence rate of patients with “W & W” is still 21-25% within 2 years (1–3), and 7% of patients with LE strategy have local recurrence within 5 years (4). Therefore, whether cCR can be used as the standard to determine the treatment strategy of patients with LARC after nCRT is still debatable. Currently, it is indisputable that pCR is the best endpoint of nCRT, Pang's meta-analysis showed that patients with pCR were superior to cCR patients in 2-year LR (0.568% *vs.* 9.82%) and 5-year prognosis (DFS: 86.86% *vs.* 73.33%, OS: 91.92% *vs.* 80%) (5). However, patients who have obtained pCR after nCRT must be confirmed by pathology after surgery, so it is necessary to find an effective method to evaluate whether patients have achieved pCR before surgery.

^18^F-fluorodeoxyglucose positron emission tomography–computed tomography (^18^ F-FDG PET-CT) can not only distinguish benign and malignant lesions accurately through the uptake of ^18^F-FDG by cells, but also has important significance in evaluating the therapeutic response of tumors. However, due to the high cost of examination, PET-CT is still mainly used in the differential diagnosis of newly diagnosed patients, and its application in evaluating the efficacy of cancer patients is relatively rare. For LARC patients, although the value of PET-CT imaging parameters in the evaluation of nCRT reactivity has been reported, due to the different intervals between the second PET-CT scaning and nCRT in these studies, the significant evaluation parameters and their optimal cut-off values are different (6–8).

In this study, we screened 52 LARC patients who had performed PET-CT scans within 1 week before nCRT and 8-9 weeks after nCRT (shortly before TME surgery) from previous clinical data, and analyzed the predictive values of PET-CT imaging parameters for GR and pCR patients. The purpose is to provide a basis for the selection of individualized treatment options for LARC patients with GR after nCRT.

## Materials and methods

### Patients

LARC patients who completed neoadjuvant therapy combined with TME surgery, and performed PET-CT scans before and after neoadjuvant therapy in the Fourth Hospital of Hebei Medical University from January 2016 to March 2020, were included in this study. The inclusion criteria were as follows: (1) Histopathology confirmed rectal adenocarcinoma before neoadjuvant therapy; (2) T3-4, N0/N+, and M0 were diagnosed by imaging examination (Chest CT, Abdominal and Pelvic MRI) at initial diagnosis; (3) Neoadjuvant therapy and TME surgery were completed before entering this study; (4)The mode of neoadjuvant therapy was long- course concurrent chemoradiotherapy recommended by NCCN guidelines; (5) Before entering this study, two PET-CT examinations were completed, and the first PET-CT was performed within 1 week before nCRT, and the second PET-CT was performed within 8-9 weeks after nCRT. The exclusion criteria included: (1) Patients with other malignancies besides rectal cancer; (2) Patients with primary tumor baseline assessment of T1-2 before nCRT; (3) The timing of PET-CT scan was not within the period required for enrollment in this study; (4) SUVmax value of the primary lesion <2.0 in the first PET-CT; (5) The neoadjuvant therapy was chemotherapy alone, short-course radiotherapy (SCRT) or induction chemotherapy before radiotherapy; (6) The second PET-CT scan revealed distant metastasis. The collection of clinical data was approved by the ethics committee of the fourth hospital of Hebei Medical University (Number: 2021104). The data are anonymous, and the requirement for informed consent was therefore waived.

### Treatment procedures

The treatment plan was determined by a multidisciplinary team consisting of gastrointestinal surgeons, medical oncologists, medical imaging specialists, and radiation oncologists. The neoadjuvant regimen was long-course concurrent chemoradiotherapy, and TME surgery was performed 9-10 weeks after nCRT. The prescription dose of radiotherapy was implemented in two phases: the first phase was 45Gy/25 fractions (45Gy/25F), 1.8Gy/1F, 5F/W, and the dose was received by planning target volume (PTV); the second phase was 5.4Gy/3F, 1.8Gy/1F, 5F/W, and the dose was received by clinical target volume-high (CTV-H). The radiotherapy targets were defined on the basis of the International Commission on Radiation Units and Measurements report no. 83 (2010) and the recommendations by Nancy in his academic writings (9). All patients received capecitabine (825 mg m^–2^) bid by oral concurrently with RT, and suspended drug use when RT was disrupted every weekend.

### PET-CT scans and image parameter analysis

The baseline PET-CT was completed within 1 week before nCRT, and the second PET-CT scan was completed within 8-9 weeks after radiotherapy. Patients were fasting for at least 6 hours before injection of ^18^F-FDG (2.6-3.7 MBq/kg), and fasting blood glucose was required to be less than 12mg/dl. PET-CT scans (GEMINI GXL-16 PET-CT scanner, Philips, Netherlands) was performed after one hour of calm rest post-injection. All scans were performed on the same scanner with the same acquisition and reconstruction protocols. Each patient was asked to empty as much urine as possible before the scan. Patient was supine with arms raised during the scan. Spiral CT scan from the top of the head to the mid-thigh. PET scanning parameters were as follows: ube voltage, 120kV; tube current, 160 mAs; matrix, 512×512; pitch 0.813, slice thickness, 5mm; and rotation time, 0.5s. The PET image was acquired at 2.5min/bed using a three-dimensional model. PET and CT images were fused with syntegra software and transmitted to the Philips service workstation.

The PET-CT images were read by two experienced radiology and nuclear medicine physicians, and the imaging parameters for the efficacy assessment of patients were as follows: SUVmax (incl. pre-SUVmax, post-SUVmax); SUVmean (incl. pre-SUVmean, post-SUVmean); metabolic tumor volume (MTV), (incl. pre-MTV, post-MTV); response index (RI), RI=[(pre-SUVmax–post-SUVmax)/pre-SUVmax]×100%; total lesion glycolysis (TLG) (incl. pre-TLG, post-TLG), TLG=SUVmean×MTV; Ratio of tumor SUVmean to liver SUVmean×100% (incl. pre-Ratio, post-Ratio).

### Pathological response evaluation

The efficacy of nCRT was evaluated by two pathologists according to the tumor regression grading (TRG) of mDworak standard, which was established by Kim (10) based on the standard of Dworak (11). The mDworak TRG standard is as follows: TRG 1 (minimal regression), defined as a dominant tumor mass encompassing more than 50% of the primary tumor and/or regional lymph node (LN) metastase; TRG 2 (moderate regression), defined as dominant fibroinflammatory changes with vasculopathy encompassing more than 50% of the entire tumor, including the tumor, regional LN metastases, and perirectal tumor deposits; TRG 3 (near complete regression), defined as one or two microscopic foci (each< 0.5 cm in diameter) of residual tumor cells or groups in the primary tumor and regional LNs; TRG 4 (complete regression), defined as no residual tumor cells in the primary tumor and regional LNs (ypT0N0). In this study, we defined TRG 3 as near-pCR, TRG 4 as pCR, TRG 3 and TRG 4 as good response (GR).

### Statistical analysis

Statistical analysis was performed using SPSS software 22.0 (SPSS, Inc., Chicago, IL, USA) and MedCalc Version 16.2. Quantitative data that conform to normal distribution were described in the form of x ± SD, while those that do not conform to normal distribution were described in the form of median (quaternary interval), i.e. M (Q_R_). Comparison of differences among quantitative data: T test was used for those meeting normal distribution and homogeneity of variance, while Wilcoxon rank sum test was used for those not meeting normal distribution. Spearman method was used to analyze the correlation between imaging parameters and tumor regression. The correlation coefficient was expressed by r_s_, 0.2< r_s_ ≤0.4 means weak correlation, 0.4≤ r_s_ <0.6 means moderate correlation, 0.6 ≤ r_s_ ≤ 1.0 means a strong correlation. Receiver operating characteristic (ROC) curve analysis was used to evaluate the diagnostic performance of significant imaging parameters. In this study, P < 0.05 was considered statistically significant.

## Results

### Characteristics of enrolled patients

**Figure 1:**
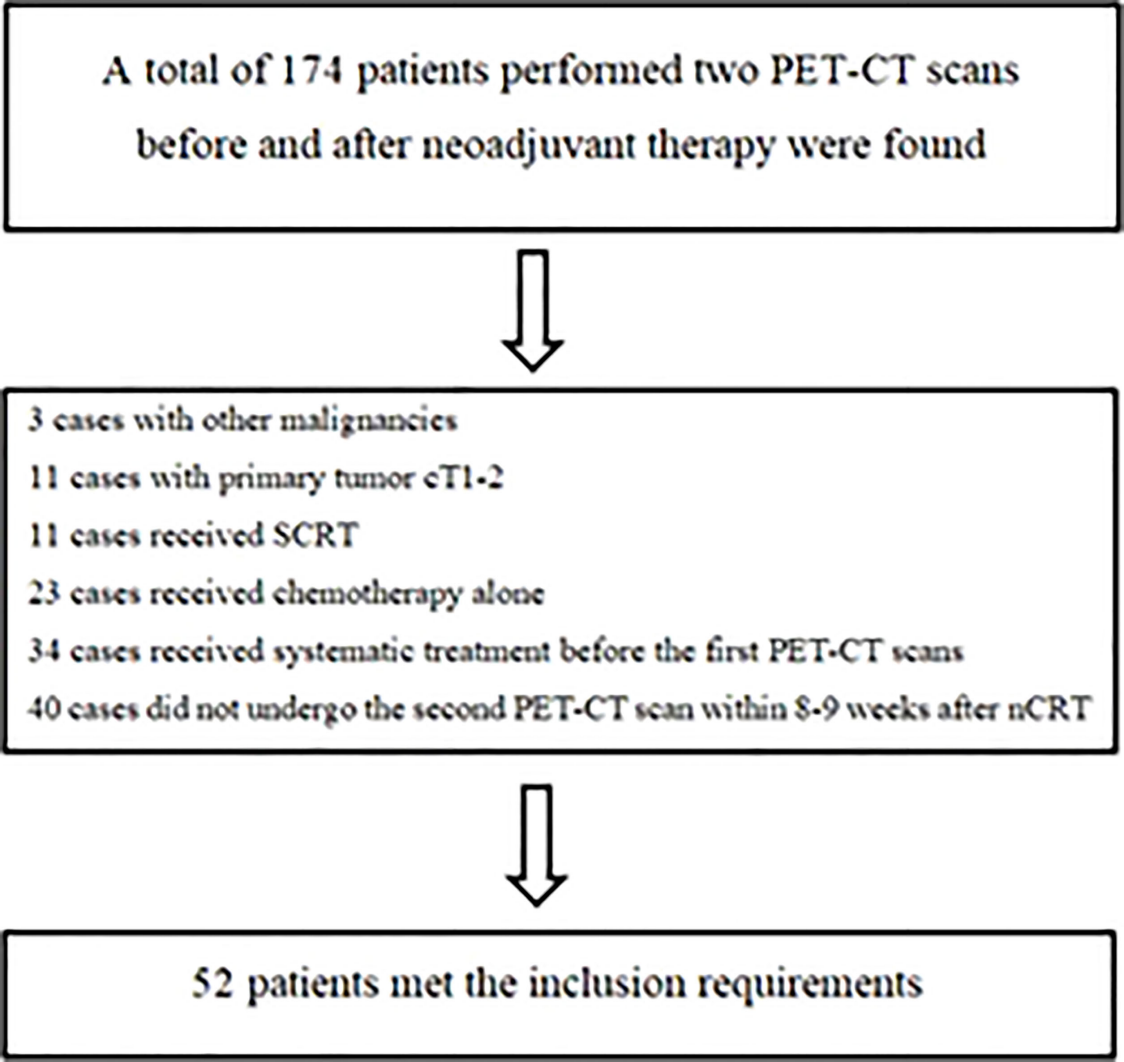
Screening process of selected patients.

From January 2016 to March 2020, there were 174 LARC patients who completed two PET-CT scans before and after neoadjuvant treatment were found in our center, of which 52 patients met the screening criteria of this study (**Figure 1**). The median age of these patients (42 males and 10 females) was 53.2 years (36-77.6), and the clinical stage was as follows: 36 patients had cT3, 16 had cT4 tumors; 4 patients had cN0, 7 had cN1 and 41 had cN2 diseases. In terms of the efficacy evaluation of nCRT, according to the mDworak standard, pathology experts identified 13 of 52 cases with TRG 1, 26 with TRG 2, 4 with TRG 3, and 9 with TRG 4. In our study, patients with TRG 1-2 status were defined as non-GR, TRG 3-4 status as GR, TRG3 status as near-pCR, TRG 4 status as pCR, and patients without TRG 4 status were defined as non-pCR. The main clinical characteristics of the patients and their response status to nCRT were listed in **Table 1**.

**Table 1:** Characteristics of patients and tumor response to nCRT (N, %).

Characteristic	near-pCR	pCR	non-pCR	GR	non-GR
Gender					
Male	3 (7.14%)	7 (16.67%)	35 (83.33%)	10 (23.81%)	32 (76.19%)
Female	1 (10.00%)	2 (20.00%)	8 (80.00%)	3 (30.00%)	7 (70.00%)
Age					
<53.2Y	2 (7.69%)	6 (23.08%)	20 (76.92%)	8 (30.77%)	18 (69.23%)
>53.2Y	2 (7.69%)	3 (11.54%)	23 (88.46%)	5 (19.23%)	21 (80.77%)
Distance to anal verge					
≤5cm	2 (10.00%)	4 (20.00%)	16 (80.00%)	6 (30.00%)	14 (70.00%)
>5cm	2 (6.25%)	5 (15.63%)	27 (84.37%)	7 (21.88%)	25 (78.12%)
T-stage					
cT_3_	2 (5.56%)	5 (13.89%)	31 (86.11%)	7 (19.44%)	29 (80.56%)
cT_4_	2 (12.50%)	4 (25.00%)	12 (75.00%)	6 (37.50%)	10 (62.50%)
N-stage					
cN_0_	0 (0.00%)	1 (25.00%)	3 (75.00%)	1 (25.00%)	3 (75.00%)
cN_1_	2 (28.57%)	1 (14.29%)	6 (85.71%)	3 (42.86%)	4 (57.14%)
cN_2_	2 (4.88%)	7 (17.07%)	34 (82.93%)	9 (21.95%)	32 (78.05%)
Total	4 (7.69%)	9 (17.31%)	43 (82.69%)	13 (25.00%)	39 (75.00%)

nCRT, neoadjuvant chemoradiotherapy; pCR, pathological complete regression; GR, good response.

### PET/CT imaging parameters of pCR and non-pCR patients

**Figure 2:**
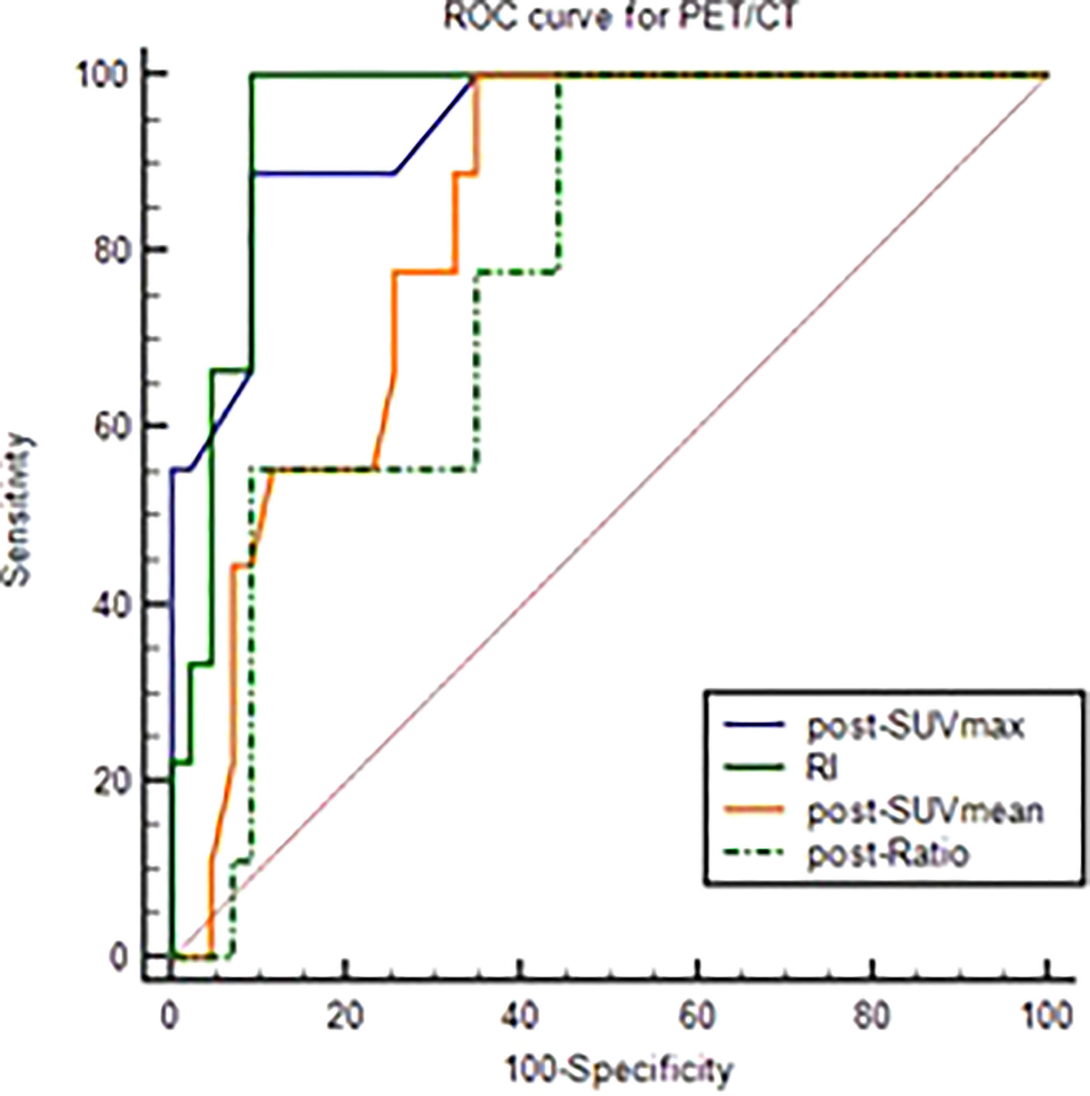
ROC curve of post-SUVmax, RI, post-SUVmean and post-Ratio using the pCR as test variable.

After analyzed the difference of PET-CT parameters between pCR and non-pCR patients by Wilcoxon rank sum test, we found that there were statistically significant differences in post-SUVmax, RI, post-SUVmean and post-Ratio between the two groups (P < 0.05). RI in pCR group was significantly higher than that in non-PCR group, while post-SUVmax, post-SUVmean and post-Ratio were significantly lower than those in non-pCR group. Spearman correlation analysis showed that RI (r_s_ = 0.59, P < 0.001) and post-SUVmax (r_s_= -0.58, P < 0.001) were near-strongly correlated with pCR, post-SUVmean (r_s_ = -0.43, P=0.001), was moderately negatively correlated with pCR, while post-Ratio (r_s_=-0.36, P=0.009) was weakly negatively correlated with pCR, as shown in **Table 2**. ROC curve analysis showed that the area under curve (AUC) of post-SUVmax, RI, post-SUVmean and post-Ratio were 0.939, 0.951, 0.831 and 0.775, respectively. Using post-SUVmax ≤ 2.5, RI > 0.67, post-SUVmean ≤ 2.2 and post-Ratio ≤ 1.39 as cut-off values, the sensitivity of these parameters to pCR diagnosis were 88.89%, 100%, 100% and 100%, the specificity were 90.7%, 90.7%, 65.12% and 55.81%, the positive predictive values (PPVs) were 66.68%, 69.24%, 37.51% and 32.14%, and the negative predictive values (NPVs) were 97.5%, 100%, 100% and 100%, respectively, as shown in **Figure 2** and **Table 3**.

**Table 2:** Correlation analysis of parameters of ^18^F-FDG PET-CT in pCR and non-pCR.

Parameters	Wilcoxon			Spearman	
	pCR, M(Q_R_)	non-pCR,M(Q_R_)	*P* value	r_s_	*P* value
Pre-SUVmax	8.50 (7.55)	7.78 (6.80)	0.371	0.13	0.367
post-SUVmax	2.10 (0.55)	6.10 (4.52)	<0.001	-0.58	<0.001
RI	0.75(0.09)	0.41(0.47)	<0.001	0.59	<0.001
pre-SUVmean	5.56(2.09)	4.52 (3.82)	0.371	0.13	0.376
post-SUVmean	1.53(0.61)	2.91(2.48)	0.002	-0.43	0.001
pre-MTV	22.00 (28.00)	28.00(22.00)	0.377	-0.12	0.382
post-MTV	4.00(12.00)	5.00(5.00)	0.567	-0.08	0.572
ΔMTV	0.77(0.18)	0.77(0.22)	0.856	0.07	0.641
pre-TLG	109.46(109.09)	139.02(140.46)	0.781	-0.04	0.784
post-TLG	6.60 (18.04)	17.25 (20.24)	0.137	-0.21	0.138
ΔTLG	0.92(0.05)	0.88(0.18)	0.064	0.03	0.821
pre-Ratio	2.79 (2.26)	2.44 (1.47)	0.461	0.10	0.466
post-Ratio	0.79 (0.50)	1.59 (1.47)	0.010	-0.36	0.009

pCR, pathological complete regression; SUVmax, the maximum standard uptake value; SUVmean, the mean standard uptake values; MTV, metabolic tumor volume; TLG, total lesion glycolysis; RI, response index; rs, Spearman's correlation coefficient; pre-, Before neoadjuvant therapy; post-, After neoadjuvant therapy; Ratio, Tumor SUVmean/ liver SUVmean.

**Table 3:** ROC analysis of PET-CT parameters to pCR.

Parameters	AUC	95%CI	Cut-off	Sensitivity	Specificity	PPV	NPV
post-SUVmax	0.939	0.836-0.987	≤2.5	88.89%	90.7%	66.68%	97.5%
RI	0.951	0.853-0.992	≥0.67	100%	90.7%	69.24%	100%
post-SUVmean	0.831	0.701-0.920	≤2.2	100%	65.12%	37.51%	100%
post-Ratio	0.775	0.638-0.879	≤1.392	100%	55.81%	32.14%	100%

pCR, pathological complete regression; SUVmax, the maximum standard uptake value; SUVmean, the mean standard uptake values; RI, response index; pre-, Before neoadjuvant therapy; post-, After neoadjuvant therapy; Ratio, Tumor SUVmean/ liver SUVmean; PPV, positive predictive value; NPV, negative predictive value.

### PET/CT imaging parameters of GR and non-GR patient

**Figure 3:**
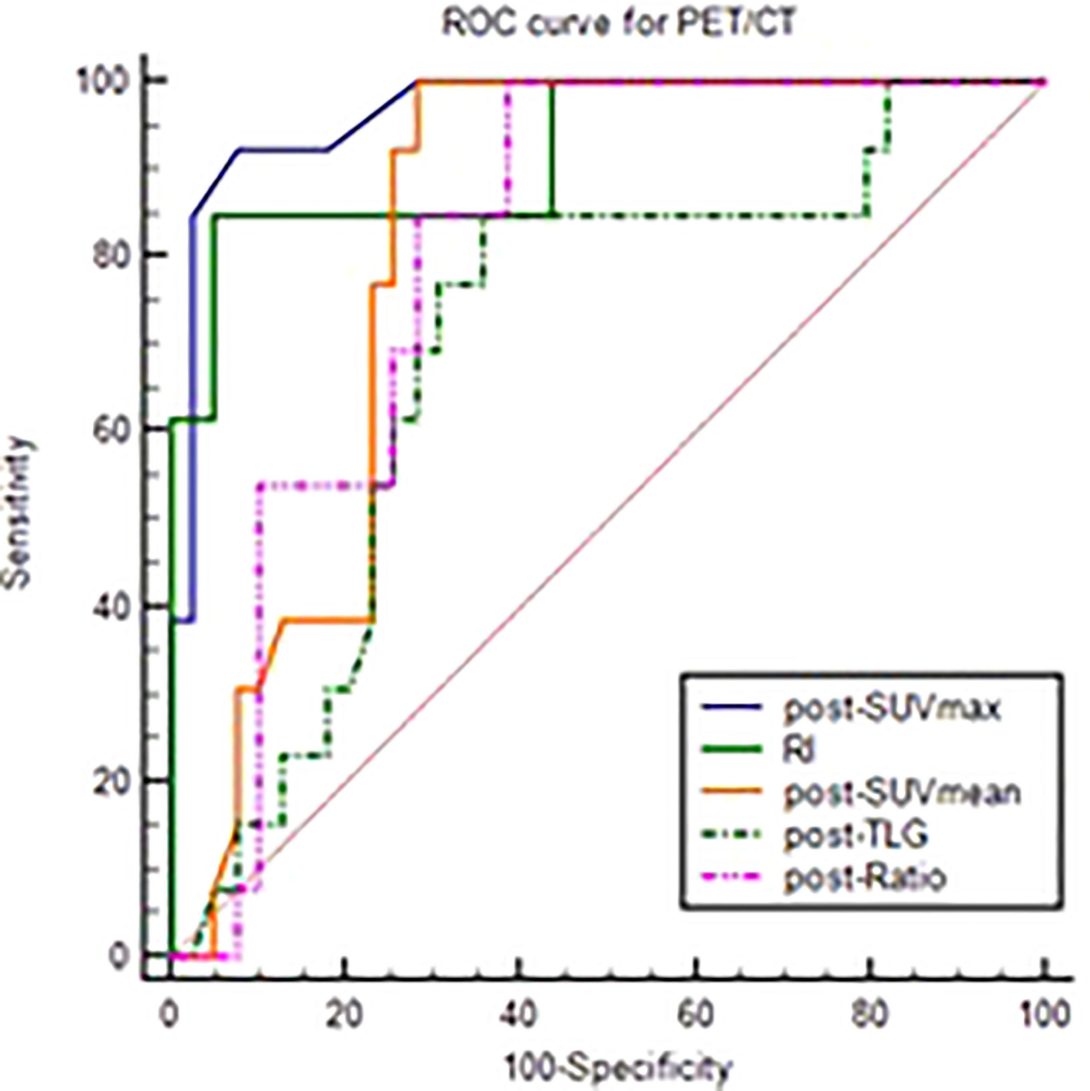
ROC curve of post-SUVmax, RI, post-SUVmean, post-TLG, post-Ratio using the GR as test variable.

The differential analysis for PET-CT parameters between GR and non-GR patients showed that there were statistically significant differences in post-SUVmax, RI, post-SUVmean, post-TLG, post-Ratio between the two groups (P< 0.05). RI in GR group was not only significantly higher than that in non-GR group, but also had a strong positive correlation with GR (r_s_=0.63, P< 0.001). The values of post-SUVmax, post-SUVmean, post-TLG and post-Ratio were significantly lower than those in non-GR group. Post-SUVmax was strongly negatively correlated with GR (r_s_=-0.70, P < 0.001), post-SUVmean and post-Ratio were moderately negatively correlated with GR (r_s_=-0.48, P< 0.001; r_s_=-0.46, P=0.009), and post-TLG was weakly negatively correlated with GR (r_s_=-0.30, P=0.032), as shown in **Table 4**. In the analysis of ROC curve, we found that AUCs of post-SUVmax, RI, post-SUVmean, post-TLG, post-Ratio were 0.966, 0.921, 0.821, 0.698 and 0.805, respectively. Using post-SUVmax≤ 2.6, RI> 0.67, post-SUVmean≤ 2.2, post-TLG≤ 11.88 and post-Ratio≤ 1.39 as cut-off values, the sensitivity of these parameters to GR diagnosis were 92.31%, 84.66%, 100%, 84.62%, and 100%, the specificity were 92.31%, 94.87%, 71.79%, 64.1%, and 61.54%, the PPVs were 80.01%, 84.61%, 54.16%, 44%, and 46.43%, and the NPVs were 97.3%, 94.87%, 100%, 92.59%, and 100%, respectively, as shown in **Figure 3** and **Table 5**.

**Table 4:** Correlation analysis of parameters of PET-CT in GR and non-GR.

Parameters	Wilcoxon			Spearman	
	GR, M(Q_R_)	non-GR,M(Q_R_)	*P* value	r_s_	*P* value
Pre-SUVmax	8.50(7.45)	7.78(6.70)	0.767	0.04	0.771
post-SUVmax	2.40(0.45)	6.20(4.50)	<0.001	-0.70	<0.001
RI	0.75(0.10)	0.38(0.45)	<0.001	0.63	<0.001
pre-SUVmean	5.56(2.58)	4.52(2.99)	0.583	0.08	0.588
post-SUVmean	1.75(0.52)	3.45(2.37)	0.001	-0.48	<0.001
pre-MTV	22.00(19.50)	28.00(21.00)	0.139	-0.21	0.140
post-MTV	4.00(2.50)	5.00(5.00)	0.282	-0.15	0.287
ΔMTV	0.77(0.28)	0.77(0.21)	0.711	0.13	0.362
pre-TLG	86.80(52.78)	144.04(169.83)	0.180	-0.19	0.182
post-TLG	7.00(6.43)	17.40(21.84)	0.034	-0.30	0.032
ΔTLG	0.92(0.06)	0.88(0.20)	0.089	0.11	0.459
pre-Ratio	2.67(2.25)	2.44(1.48)	0.4907	-0.02	0.909
post-Ratio	0.83(0.31)	1.62(1.52)	0.001	-0.46	0.001

GR, good response; SUVmax, the maximum standard uptake value; SUVmean, the mean standard uptake values; MTV, metabolic tumor volume; TLG, total lesion glycolysis; RI, response index; rs, Spearman's correlation coefficient; pre-, Before neoadjuvant therapy; post-, After neoadjuvant therapy; Ratio, Tumor SUVmean/ liver SUVmean.

**Table 5:** ROC analysis of PET-CT parameters to GR.

	AUC	95%CI	Cut-off	Sensitivity	Specificity	PPV	NPV
post-SUVmax	0.966	0.875-0.997	≤2.6	92.31%	92.31%	80.01%	97.3%
RI	0.921	0.812-0.978	≥0.67	84.62%	94.87%	84.61%	94.87%
post-SUVmean	0.821	0.689-0.913	≤2.2	100%	71.79%	54.16%	100%
post-TLG	0.698	0.555-0.818	≤11.88	84.62%	64.10%	44%	92.59%
post-Ratio	0.805	0.671-0.902	≤1.392	100%	61.54%	46.43%	100%

GR, good response; SUVmax, the maximum standard uptake value; SUVmean, the mean standard uptake values; RI, response index; TLG, total lesion glycolysis; pre-, Before neoadjuvant therapy; post-, After neoadjuvant therapy; Ratio, Tumor SUVmean/ liver SUVmean; PPV, positive predictive value; NPV, negative predictive value.

## Discussion

Currently, as the standard treatment for LARC patients, nCRT has been widely used in clinical practice. However, not all patients can benefit from this treatment. Studies show that only about 20% of patients can achieve pCR efficacy (12–15). In this study, we evaluated the efficacy of 52 patients, and found that 17.31% of patients obtained pCR and 25.00% obtained GR (pCR+ near-pCR). As we all know, the ideal goal of tumor treatment is to enable patients to obtain “no evidence of disease” status ( NED) and long-term survival. The complete response (CR) status is the assessment of good therapeutic efficacy of tumor patients and is the interpretation of NED, including clinical complete response (cCR) and pCR. For LARC patients, cCR is a dynamic concept with time limit, obtained based on enteroscopy, imaging and other data, since the results of cCR obtained through existing examination methods may be denied by a more advanced examination result in the future. Studies have shown that the local recurrence rate of two years of LARC patients obtained cCR is still over 20% after “W&W” strategy was adopted (1–3). However, pCR and near-pCR are postoperative pathological results, which are outcome concepts and have a more critical impact in prognosis. Studies have shown that only 36% patients assessed as cCR in LARC patients after completing nCRT can be verified for pCR (16). Therefore, exploring a method that can accurately predict the responsiveness of LARC patients to neoadjuvant therapy is of great significance for guiding clinicians to choose the follow-up treatment after neoadjuvant therapy.

It is well known that ^18^F-FDG PET-CT has high sensitivity and specificity in the diagnosis of malignant tumors. Up to now, a number of studies have reported the significance and application value of PET-CT imaging parameters in predicting LARC patients after receiving nCRT. However, due to the inconsistency of PET-CT scanning timing after nCRT, the predicted values of various imaging parameters are not consistent in clinical application. In our study, the timing of the second PET-CT scan was set within 1 week before surgery, that is, 8-9 weeks after nCRT, to better reflect the tumor status pre-operation. And our results show that post-SUVmax, RI, post-SUVmean and post-TLG are of high value in evaluating the status of pCR status of LARC patients, and post-ratio was also a good predictor of GR in addition to the above indicators.

Post-SUVmax and post-SUVmean are parameters that reflect the uptake capacity of ^18^F-FDG by tumor cells after receiving nCRT. In this study, we found that these two parameters were significantly lower in patients who obtained pCR than in non-pCR patients. When the Cut-off values of post-SUVmax ≤2.5, its NPV was as high as 97.5%, that is, only 2.5% of the patients who obtained pCR using this parameter might be mistaken for non-pCR. Similarly, when the Cut-off values of post-SUVmean ≤2.2, its NPV was up to 100%. For GR and non-GR, these two parameters also had a good exclusion diagnostic advantage, with a NPV of 97.3% for post-SUVmax at Cut-off ≤2.6, and 100% for post-SUVmean at Cut-off ≤ 2.2. The NPV and PPV of post-SUVmax obtained by Mafione et al. in predicting GR were 69.56% and 91.30%, respectively (we obtained by further calculations based on the information provided in the original paper). This result is somewhat different from our study, which may be due to the fact that Maffione's second PET-CT was performed 4.6-17.4 weeks after nCRT (17). In another study (18), the researchers set the timing of the second PET-CT at 6-7 weeks after the completion of nCRT and found that the sensitivity, specificity, PPV and NPV of post-SUVmax in predicting pCR were 87.5%, 34.4%, 25% and 91.7% respectively. The advantage of this result in negative predictive value was similar to our results, which also reflected the advantage of post-SUVmax in excluding false-positive patients.

MTV and TLG are two parameters that reflect the overall glucose metabolism of tumor. MTV mainly reflects tumor metabolic volume, which is a direct parameter obtained after PET-CT scanning, while TLG is an indirect parameter obtained by MTV and SUVmean, which reflects both metabolic volume and metabolic activity of tumor cells (19). It is found that TLG is of great value in the prognosis of patients with non-small cell lung cancer, renal cell carcinoma, head and neck cancer, ovarian cancer and soft tissue sarcoma (20–24). In this study, we found that post-TLG had a negative predictive value of 92.59% for GR status in LARC patients, but did not show a predictive advantage in pCR status. In previous studies, there have been meaningful reports about MTV, ΔMTV and ΔTLG in predicting the responsiveness of LARC patients to nCRT (8, 25–27). However, in our study, none of these three parameters was found to have meaningful predictive value in our study. Therefore, the predictive value of these parameters needs to be further explored.

The RI value reflects the change of an ability of tumor to uptake ^18^F-FDG before and after therapy, which is also described as ΔSUVmax, Δ%SUV or ΔSUVmax% in some literatures. Murcia et al. found that the RI value was 79.9±4.69% in the responders, significantly higher than 60.3±4.6% in the non-responders, suggesting that this parameter has important value in differentiating the response ability of LARC patients to nCRT (28). In the study of leccisotti et al., patients received three time PET-CT scans in different timing (before nCRT, at the end of the second week of nCRT, and shortly before surgery), obtained early RI and late RI, and found that the early RI had a high predictive value for the pCR status. When the cut-off value was 61.2%, the sensitivity, specificity, PPV and NPV were 85.4%, 65.2%, 90% and 56%, respectively (7). In terms of predicting GR status, Capirci's study showed that when cut-off value was 63.4%, the predictive sensitivity, specificity, PPV, NPV and overall accuracy of RI for GR were 84.5%, 80%, 81.4%, 84.2% and 81% respectively, and the AUC of ROC curve was 0.862 (29). Caruso et al. found that when cut-off values were 70%, the sensitivity, specificity, PPV and NPV of Δ%SUV for GR prediction were 84.4%, 80%, 81.4% and 84.2%, respectively (30). In this study, we conducted ROC analysis on the predictive value of RI and found that AUCs obtained by pCR and GR were 0.951 and 0.921, respectively. When cut-off values were 67%, the sensitivity, specificity, PPV and NPV for pCR were 100%, 90.7%, 69.24% and 100%, respectively, and 84.62%, 94.87%, 84.61% and 94.87% for GR. It can be seen from previous results and our study that RI value is of high value in predicting whether LARC patients can obtain pCR and GR after receiving nCRT, and RI value of 60-70% or higher indicates high reactivity of patients to nCRT.

In this study, the timing of two PET-CT scans was strictly limited when the patients were enrolled. In particular, the time of the second scans were limited to in the 8 to 9 weeks after the completion of nCRT. This is exactly coincided with the timing of TME surgery recommended by the current diagnosis and treatment guidelines, and also reflects the status of the specimens *in vivo* to the greatest extent. However, this also leads to the limitation of number of cases in this study. Therefore, in subsequent clinical practice, we will increase the sample size to verify our results. In addition, as most patients receiving neoadjuvant therapy in our center chose a long course of nCRT in terms of treatment mode, we did not analyze patients receiving SCRT in this study, which might also be a limitation. Furthermore, in the inclusion criteria, the number of chemotherapy cycles between long-course nCRT and TME surgery was not limited, so the relationship between whether patients were treated with total neoadjuvant therapy (TNT) mode and the tumor reactivity of patients' PET-CT imaging parameters was not analyzed. Due to the TNT treatment model has been preferentially recommended by the NCCN guidelines since 2022, the prediction of efficacy by PET-CT has not yet been reported. It will be a new direction to study the prediction of tumor reactivity in TNT mode.

Under the premise of strictly limiting the inclusion criteria, this study obtained the predictive value of ^18^F-FDG PET-CT imaging parameters such as post-SUVmax, post-suvmean, RI, post-Ratio and post-TLG for the tumor pathological status of LARC patients before surgery. Among these indicators, post-SUVmax and RI, which can reflect the change of the maximum uptake capacity of ^18^F-FDG by tumor tissue, are of great value in predicting the GR and pCR status of patients. The application of these parameters will provide an important reference for the selection of subsequent treatment strategies for patients with high response to nCRT.

## Data availability statement

The original contributions presented in the study are included in the article/supplementary material. Further inquiries can be directed to the corresponding author.

## Ethics statement

The studies involving human participants were reviewed and approved by the fourth hospital of Hebei Medical University. Written informed consent for participation was not required for this study in accordance with the national legislation and the institutional requirements.

## Author contributions

Conceived and designed the study: GW, XMZ. Performed the study and analyzed the data: FW, XXZ, KW, CY, CZ. Wrote the paper: XXZ, FW, LX. Supervised the entire study and review the final paper: GW, XMZ, XXZ, FW, KW, CZ, CY, LX. All authors contributed to the article and approved the submitted version.

## References

[1] van der Valk MJM Hilling DE Bastiaannet E Meershoek-Klein Kranenbarg E Beets GL . Long-term outcomes of clinical complete responders after neoadjuvant treatment for rectal cancer in the international watch & wait database (IWWD): an international multicentre registry study. Lancet. (2018) 391(10139):2537–45. doi: 10.1016/S0140-6736(18)31078-X 29976470

[2] Chadi SA Malcomson L Ensor J Riley RD Vaccaro CA Rossi GL . Factors affecting local regrowth after watch and wait for patients with a clinical complete response following chemoradiotherapy in rectal cancer (InterCoRe consortium): an individual participant data meta-analysis. Lancet Gastroenterol Hepatol (2018) 3(12):825–36. doi: 10.1016/S2468-1253(18)30301-7 30318451

[3] Smith JJ Strombom P Chow OS Roxburgh CS Lynn P Eaton A . Assessment of a watch-and-wait strategy for rectal cancer in patients with a complete response after neoadjuvant therapy. JAMA Oncol (2019) 5(4):e185896. doi: 10.1001/jamaoncol.2018.5896 30629084 PMC6459120

[4] Rullier E Vendrely V Asselineau J Rouanet P Tuech JJ Valverde A . Organ preservation with chemoradiotherapy plus local excision for rectal cancer: 5-year results of the GRECCAR 2 randomised trial. Lancet Gastroenterol Hepatol (2020) 5(5):465–74. doi: 10.1016/S2468-1253(19)30410-8 32043980

[5] Pang K Rao Q Qin S Jin L Yao H Zhang Z . Prognosis comparison between wait and watch and surgical strategy on rectal cancer patients after treatment with neoadjuvant chemoradiotherapy: a meta-analysis. Therap Adv Gastroenterol (2019) 12:1756284819892477. doi: 10.1177/1756284819892477 PMC6891008 31832099

[6] Koç M Kaya GÇ Demir Y Sürücü E Sarioğlu S Obuz F . The value of liver-based standardized uptake value and other quantitative 18F-FDG PET-CT parameters in neoadjuvant therapy response in patients with locally advanced rectal cancer: correlation with histopathology. Nucl Med Commun (2015) 36(9):898–907. doi: 10.1097/MNM.0000000000000342 25969176

[7] Leccisotti L Gambacorta MA de Waure C Stefanelli A Barbaro B Vecchio FM . The predictive value of 18F-FDG PET/CT for assessing pathological response and survival in locally advanced rectal cancer after neoadjuvant radiochemotherapy. Eur J Nucl Med Mol Imaging. (2015) 42(5):657–66. doi: 10.1007/s00259-014-2820-9 25687534

[8] Avallone A Aloj L Pecori B Caracò C De Stefano A Tatangelo F . (18)F-FDG PET/CT is an early predictor of pathologic tumor response and survival after preoperative radiochemotherapy with bevacizumab in high-risk locally advanced rectal cancer. J Nucl Med (2019) 60(11):1560–8. doi: 10.2967/jnumed.118.222604 PMC6836863 30877175

[9] Nancy YL Jiade JL . Target volume delineation and field setup: a practical guide for conformal and intensity-modulated radiation therapy. Berlin, Heidelberg: Springer-Verlag (2013) p. 161–8. doi: 10.1007/978-3-642-28860-9

[10] Kim SH Chang HJ Kim DY Park JW Baek JY Kim SY . What is the ideal tumor regression grading system in rectal cancer patients after preoperative chemoradiotherapy? Cancer Res Treat (2016) 48(3):998–1009. doi: 10.4143/crt.2015.254 26511803 PMC4946373

[11] Dworak O Keilholz L Hoffmann A . Pathological features of rectal cancer after preoperative radiochemotherapy. Int J Colorectal Dis (1997) 12(1):19–23. doi: 10.1007/s003840050072 9112145

[12] Ferrari L Fichera A . Neoadjuvant chemoradiation therapy and pathological complete response in rectal cancer. Gastroenterol Rep (Oxf). (2015) 3(4):277–88. doi: 10.1093/gastro/gov039 PMC4650974 26290512

[13] Tawfik B Mokdad AA Patel PM Li HC Huerta S . The neutrophil to albumin ratio as a predictor of pathological complete response in rectal cancer patients following neoadjuvant chemoradiation. Anticancer Drugs (2016) 27(9):879–83. doi: 10.1097/CAD.0000000000000411 27434664

[14] Maas M Nelemans PJ Valentini V Das P Rödel C Kuo LJ . Long-term outcome in patients with a pathological complete response after chemoradiation for rectal cancer: a pooled analysis of individual patient data. Lancet Oncol (2010) 11(9):835–44. doi: 10.1016/S1470-2045(10)70172-8 20692872

[15] Martin ST Heneghan HM Winter DC . Systematic review and meta-analysis of outcomes following pathological complete response to neoadjuvant chemoradiotherapy for rectal cancer. Br J Surg (2012) 99(7):918–28. doi: 10.1002/bjs.8702 22362002

[16] Cercek A Roxburgh CSD Strombom P Smith JJ Temple LKF Nash GM . Adoption of total neoadjuvant therapy for locally advanced rectal cancer. JAMA Oncol (2018) 4(6):e180071. doi: 10.1001/jamaoncol.2018.0071 29566109 PMC5885165

[17] Maffione AM Ferretti A Grassetto G Bellan E Capirci C Chondrogiannis S . Fifteen different 18F-FDG PET/CT qualitative and quantitative parameters investigated as pathological response predictors of locally advanced rectal cancer treated by neoadjuvant chemoradiation therapy. Eur J Nucl Med Mol Imaging. (2013) 40(6):853–64. doi: 10.1007/s00259-013-2357-3 23417501

[18] Martoni AA Di Fabio F Pinto C Castellucci P Pini S Ceccarelli C . Prospective study on the FDG–PET/CT predictive and prognostic values in patients treated with neoadjuvant chemoradiation therapy and radical surgery for locally advanced rectal cancer. Ann Oncol (2011) 22(3):650–6. doi: 10.1093/annonc/mdq433 20847032

[19] Larson SM Erdi Y Akhurst T Mazumdar M Macapinlac HA Finn RD . Tumor treatment response based on visual and quantitative changes in global tumor glycolysis using PET-FDG imaging. Visual response score Change total lesion glycolysis. Clin Positron Imaging. (1999) 2:159–71. doi: 10.1016/S1095-0397(99)00016-3 14516540

[20] Akhurst T Ng V Larson SM O’Donoghue JA O’Neel J Erdi Y . T Tumor burden assessment with positron emission tomography with [18-f] 2-fluoro 2-deoxyglucose (FDG PET) modeled in metastatic renal cell cancer. Clin Positron Imaging. (2000) 3(2):57–65. doi: 10.1016/s1095-0397(00)00041-8 10838401

[21] Zhang H Wroblewski K Liao S Kampalath R Penney BC Zhang Y . Prognostic value of metabolic tumor burden from (18)FFDG PET in surgical patients with non-small-cell lung cancer. Acad Radiol (2013) 20:32–40. doi: 10.1016/j.acra.2012.07.002 22999369

[22] La TH Filion EJ Turnbull BB Chu JN Lee P Nguyen K . Metabolic tumor volume predicts for recurrence and death in headand-neck cancer. Int J Radiat Oncol Biol Phys (2009) 74:1335–41. doi: 10.1016/j.ijrobp.2008.10.060 PMC2752334 19289263

[23] Chung HH Kwon HW Kang KW Park NH Song YS Chung JK . Prognostic value of preoperative metabolic tumor volume and total lesion glycolysis in patients with epithelial ovarian cancer. Ann Surg Oncol (2012) 19:1966–72. doi: 10.1245/s10434-011-2153-x 22124757

[24] Choi ES Ha SG Kim HS Ha JH Paeng JC Han L . Total lesion glycolysis by 18F-FDG PET/CT is a reliable predictor of prognosis in soft-tissue sarcoma. Eur J Nucl Med Mol Imaging. (2013) 40(12):1836–42. doi: 10.1007/s00259-013-2511-y 23880967

[25] Deantonio L Caroli A Puta E Ferrante D Apicella F Turri L . Does baseline [18F] FDG-PET/CT correlate with tumor staging, response after neoadjuvant chemoradiotherapy, and prognosis in patients with rectal cancer? Radiat Oncol (2018) 13(1):211. doi: 10.1186/s13014-018-1154-3 30359275 PMC6202838

[26] Fernando S Lin M Pham TT Chong S Ip E Wong K . Prognostic utility of serial 18F-FDG-PET/CT in patients with locally advanced rectal cancer who underwent tri-modality treatment. Br J Radiol (2020) 93(1105):20190455. doi: 10.1259/bjr.20190455 31617737 PMC6948083

[27] Pyo DH Choi JY Lee WY Yun SH Kim HC Huh JW . A nomogram for predicting pathological complete response to neoadjuvant chemoradiotherapy using semiquantitative parameters derived from sequential PET/CT in locally advanced rectal cancer. Front Oncol (2021) 11:742728. doi: 10.3389/fonc.2021.742728 34676170 PMC8523984

[28] Murcia Duréndez MJ Frutos Esteban L Luján J Frutos MD Valero G Navarro Fernández JL . The value of 18F-FDG PET/CT for assessing the response to neoadjuvant therapy in locally advanced rectal cancer. Eur J Nucl Med Mol Imaging. (2013) 40(1):91–7. doi: 10.1007/s00259-012-2257-y 23081822

[29] Capirci C Rubello D Pasini F Galeotti F Bianchini E Favero GD . The role of dual-time combined 18-fluorodeoxyglucose positron emission tomography and computed tomography in the staging and restaging workup of locally advanced rectal cancer, treated with preoperative chemoradiation therapy and radical surgery. Int J Radiat Oncol Biol Phys (2009) 74(5):1461–9. doi: 10.1016/j.ijrobp.2008.10.064 19419820

[30] Caruso R Vicente E Quijano Y Duran H Fabra I Diaz E . Role of 18F−PET−CT to redict pathological response after neoadjuvant treatment of rectal cancer. Discovery Oncol (2021) 12(1):16. doi: 10.1007/s12672-021-00405-w PMC8777577 35201442

